# Coronary heart disease in Indians: Implications of the INTERHEART study

**DOI:** 10.4103/0971-5916.73396

**Published:** 2010-11

**Authors:** Vamadevan S. Ajay, Dorairaj Prabhakaran

**Affiliations:** *Centre for Chronic Disease Control, New Delhi, India*

**Keywords:** Community, coronary heart disease, Indians, INTERHEART study, policy, prevention

## Abstract

Coronary heart diseases (CHD) have reached epidemic proportions among Indians. The recently concluded INTWERHEART study emphasizes the role of behavioural and conventional risk factors in the prediction of CHD risk among Indians. These findings have implication for the health care providers and policy makers in the country due to the fact that all these conventional risk factors are potentially modifiable and are good starting points for prevention. The policy measures by means of legislation and regulatory approaches on agriculture and food industry or tobacco or physical activity will have large impact on CHD risk factor reduction in the population. In addition, the health system needs to focus on: (*i*) providing information for increasing awareness and an enabling environment for adoption of healthy living habits by the community; (*ii*) early detection of persons with risk factors and cost-effective interventions for reducing risk; and (*iii*) early detection of persons with clinical disease and cost-effective secondary prevention measures to prevent complications. The evidence from INTERHEART provides rationale for developing treatment algorithms and treatment guidelines for CHD at various levels of health care. In addition, INTERHEART provides answer for the quest for a single reliable biomarker, Apo B/ApoA 1 ratio that can predict the future CHD risk among individuals. Further to this, the INTERHEART study also opens up several unanswered questions on the pathobiology of the premature onset of myocardial infarction among Indians and calls for the need to developing capacity in clinical research in CHD in India.

## Introduction

India is undergoing a rapid health transition with rising burden of coronary heart disease (CHD)^1^. Among adults over 20 yr of age, the estimated prevalence of CHD is around 3–4 per cent in rural areas and 8–10 per cent in urban areas, representing a two-fold rise in rural areas and a six-fold rise in urban areas between the years 1960 and 2000^2^. Studies among Indian migrants in various parts of the world have documented an increased susceptibility to CHD in comparison to the native population studied^3^,^4^. In Britain, comparisons of age adjusted mortality statistics have shown a 40–60 per cent excess in South Asian migrants (comprising Indian) in comparison to the population of England and Wales^5^. Several factors such as genetic, metabolic, early-life, conventional and non-conventional risk factors were suspected to cause high CHD morbidity and mortality rates among Indians. However, the results from the INTERHEART study conclusively established the role of behavioural and conventional risk factors in the prediction of CHD risk among Indians^6^. In this report we summarize the results of the INTERHEART study and discuss its implications for India.

## The INTERHEART study

The INTERHEART study, an international case-control study, carried out in 52 countries involving 15152 cases of incident acute myocardial infarction (AMI) and 14820 controls, estimated the hazard ratios and population-attributable fractions for multiple well-established physiological and behavioural risk factors for incident myocardial infarction in several regions of the world^6^. This study concluded that abnormal lipids, smoking, hypertension, diabetes, abdominal obesity, psychosocial stress, decreased consumption of fruits and vegetables, moderate consumption of alcohol, and physical activity accounted for most of the risk of myocardial infarction worldwide. Collectively, these nine risk factors accounted for 90 per cent of the population attributable risk (PAR) in men and 94 per cent in women. The risk of heart attacks imposed by these risk factors was similar in both sexes, for all the population groups studied at all ages in all regions emphasizing the role of environmental origin of cardiovascular risk factors for all the ethnicities of the world. The key results from the South Asian Component of the INTERHEART study are shown in the Table. The only difference observed for South Asian population was the earlier occurrence of AMI^6^. But this was explained by the higher level of risk factors particularly smoking and diabetes among Asians.

**Table:** Key results of the South Asian component of the INTERHEART study^17^

Deaths due to acute myocardial infarction (AMI) in south Asians occur at 5-10 years earlier than western population. South Asian men encountering AMI were 5.6 yr younger than women. The higher risk for AMI in South Asians in their younger age is largely determined by the higher levels of risk factors and the nine conventional risk factors (abnormal lipids, smoking, hypertension, diabetes, abdominal obesity, psychosocial factors, consumption of fruits & vegetables, alcohol and regular physical activity) collectively explain 86 per cent of the AMI risk in south Asians. Abnormal Apo-B/ApoA-1 ratio and smoking are the most important risk factors. Low education level is associated with increased risk of AMI worldwide. Protective lifestyle factors such as leisure time physical activity and regular intake of fruits and vegetables are markedly lower among south Asians than western population, while harmful risk factors such as elevated ApoB/Apo A-1 ratio are higher in south Asians. South Asians have significantly higher population attributable risk associated with waist-hip ratio. Higher level of risk factors in both cases and controls under the age of sixty. Regular alcohol consumption is not protective for AMI in south Asians (OR=1.06; 95% CI, 0.85–1.20).

## General remarks on the implications of INTERHEART

The results of INTERHEART dispelled several myths that were prevailing prior to the study. Studies in the earlier 70’s, such as the North Karelia Projects, estimated that only 50 per cent of the risk for myocardial infarction could be explained by conventional risk factors^7^. Subsequently it was estimated that 75 per cent of the population attributable risk could be explained by such risk factors^8^. Further, simple conventional risk factors were considered to be unimportant in a number of developing countries particularly in South Asians. This was largely because of the early occurrence of CHD and excess deaths among south Asians in a relatively younger population. Therefore it was believed that non-conventional risk factors and genetic factors may play a pivotal role in determining the risk of CHD among south Asians. However, the results of INTERHEART study when considered with the results of a modelled data from cohort studies dispel this notion^9^. One important observation in the INTERHEART study was the high level of risk factors even among controls who were less than sixty years of age. This observation of a larger role for conventional risk factors in increasing the risk for AMI, has important implications for Indians.

## Implications for India

Epidemiological studies (largely cross-sectional surveys) from various parts of India have reported the rising trends and a high burden in the levels of conventional risk factors such as diabetes, hypertension and metabolic syndrome which are largely determined by urbanization as evident from the urban-rural difference in the risk factors observed in India^10^–^12^. Further, the long-term case fatality following acute coronary syndrome is considerably higher among Indians as compared to other populations^13^. In addition, a reversal of socio-economic gradients for CHD risk factors has emerged in the Indian population^14^,^15^. This combined with lack of support mechanisms for evidence based treatment and follow up for AMI, results in almost 49.1 per cent higher mortality among the poor as compared to richest^16^. All these factors in combination lead to a large economic loss to India. The WHO has estimated that India lost 9 billion USD in national income from premature deaths due to heart disease, stroke and diabetes in the year 2005^17^. These losses are expected to cumulatively lead to 237 billion USD over the next 10 years^17^. These estimates highlight the need for aggressive strategies for the prevention and control of CHD in India. In this context, findings from the south Asian component of INTERHEART study convey important messages to the health care providers and policy makers in the country as all these conventional risk factors for CHD are potentially modifiable and are good starting points for prevention^18^.

## Policy implications

The positive impact of policy measures on chronic diseases is unequivocal^19^. INTERHEART study results highlight the need for policies to promote primordial prevention strategies in India such as regulation against tobacco use and promotion of protective lifestyle factors such as leisure time physical activity and regular intake of fruits and vegetables which are markedly lower among South Asians compared to western population.

Considering the size of this public health challenge, the interventions can only be addressed through policy measures by means of legislation and regulatory approaches on agriculture and food industry (production, pricing, labelling) or tobacco (production, sale, advertising) or physical activity (a conducive transport policy which favours urban cycle lanes, walking paths with curbs on private vehicular transport, facilities for leisure time exercise in community playgrounds and emphasising the importance of physical activity in school curriculum and at worksites) that have large impact on the mean level CHD risk factors at the population level. For example, in low and middle income countries, a 10 per cent increase in the price of tobacco products reduces the number of smokers by 37.6 million (9.3 million deaths averted) as compared to 18.6 million (4.4 million deaths averted) with other tobacco control measures^20^. By contrast, in high income countries, a similar intervention will reduce the number of smokers by 4.1 million (1 million deaths averted) as compared with 4.0 million (0.9 million deaths averted) by non-price measures^20^. Therefore, active health policy measures are likely to have a larger impact in developing countries such as India.

## Implications for the community

Public health education programmes focussing on promotion of (*i*) a health promoting diet (calories appropriate to the level of physical activity; moderation in the intake of saturated fat, salt, and refined sugar; high intake of fresh fruit and vegetables; fish in preference to red meat in non-vegetarian diets), and (*ii*) adequate physical activity and regular exercise are required. Similarly anti-tobacco messages are a major public health imperative which will provide the largest benefit for CHD prevention. These messages need to be reinforced to change individual, family, and community behaviours throughout the country. Such measures bring about benefits not only for CHD prevention but also for a wide range of chronic diseases such as stroke, diabetes and some cancers. The success of such population based interventions, addressing multiple risk factors for CHDs, through lifestyle linked community programmes was demonstrated initially in North Karelia study^21^. However, a meta-analysis questioned its utility^22^. In this meta-analysis Ebrahim *et al* suggested that the benefits by such interventions are very minimal and there may be small reduction in blood pressure, blood glucose and lipids. They questioned the validity of such small reduction in risk factors. However, as argued by the authors, the reasons for such miniscule reductions are several. These include strong secular trends, limitations of implementing interventions at community level, greater availability of highly cost-effective drugs to control high blood pressure and lower blood cholesterol (making high risk intervention strategies more effective as opposed to health promotion), difficulty of measuring population-level changes, methodological and statistical flaws and lack of a conceptual model.

We, however, believe that in developing countries such as India such measures may indeed work due to several reasons. First, the risk factor levels are high among Indians conferring a higher risk. Interventions are likely to have a higher impact on high risk population. Further, most studies carried out in western population were conducted in a background of declining trend of CVD, thereby negating the expected benefits (Fig.). Evidence from other developing countries suggests that public health interventions in developing countries are successful^23^. For example, the ‘Healthy lifestyle programmes’ in Mauritius had a positive impact on mean population cholesterol level, hypertension prevalence, tobacco and alcohol use^24^. In addition, we have recently demonstrated the utility of such programmes in Indian Industrial population^24^. Given that just two simple factors (tobacco and Apo B/Apo A-I ratio) explain more than two third of risk, targeting tobacco, diet and physical activity may have huge implications for the Indian population.

**Fig:**
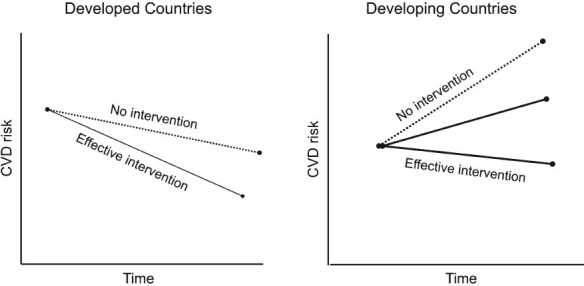
Comparison of the likely impact of population based coronary heart disease interventions between developed countries and developing countries. *Figure note*: With increasing incidence of coronary heart disease, interventions are likely to be effective as opposed to developed countries where interventions were carried when a decline in secular trends were observed.

## Implication for the health system

In the coming years, the Indian health system has to device CHD prevention and control strategies, as a priority, to address the growing epidemic of CHD. Deriving inputs from the INTERHEART results, the principal functions of such strategies would be: (*i*) to provide information and an enabling environment for increasing awareness and adoption of health living habits by the community; (*ii*) early detection of persons with risk factors and cost-effective interventions for reducing risk; and (*iii*) early detection of persons with clinical disease and cost-effective secondary prevention measures to prevent complications.

Many of these activities can be performed in primary care settings through the multi-purpose health workers (MPWs) and other non-physician health workers. MPW cadre can be trained to promote simple messages on promoting fruits and vegetable intake, physical activity and tobacco cessation. Further, simple screening tool for diabetes and CHD can be used identifying early detection of high risk individuals. For example, we and others have devised screening systems for using non-biochemical, simple measures for identifying individuals with diabetes^25^–^27^. Such risk scores can be used for targeted screening of individuals. Such tools can be easily employed by lay persons in the community enabling self-referral.

Addition of fasting values of blood fats and blood sugar to the above tools will substantially improve their specificity and can be employed at the secondary health care level. Additionally, the WHO STEPwise approach to surveillance methodology can be adopted for surveillance of risk factors which is adapted for low resource settings^28^. Thus, the recently launched National Programme for Prevention and Control of Diabetes, Cardio-vascular Diseases and Stroke (NPCDS) can derive substantial inputs from the INTERHEART study while devising its strategies. Community-based programmes targeting multiple risk factors impact blood pressure much more than other risk factors^29^. If a comprehensive risk factor survey is not possible, even prevention programmes aimed at controlling high blood pressure could be a starting point because of its significant effect on death rates from heart disease and stroke and complications among diabetic cases.

## Implications for physicians

For primary care physicians and cardiologists, INTERHEART study provides ample evidence to give simple messages on the role of conventional risk factors in the causation of heart attacks and convincing the patients on the need for adopting healthy lifestyle for primary and secondary prevention of heart attack has become straight forward. Above all, INTERHEART provides answer for the quest for a single reliable biomarker, the Apo B/ApoA 1 ratio that can predict the future CHD risk relieving physicians from the complexities of monitoring various fractions of blood fats and other conventional and unconventional risk factors. Aggressive management of coronary risk factors in South Asians entails vigorous lipid control, and dietary and lifestyle modifications. The evidence from INTERHEART provides rationale for developing treatment algorithms and treatment guidelines for CHD at various levels of health care. Further, this approach improves quality of care and ensures equity as the intervention tools do not involve complex and costly therapeutic procedures.

## Unanswered questions

While explaining the role of conventional risk factors in the aetiology of myocardial infarction, INTERHEART study also opens up several unanswered questions such as:

- Why do Indians have higher levels of risk factors even at a younger age?
- Why is that CHD/ diabetes occurs at younger ages and at a lower level of risk factors?
- Are these factors a result of gene-environment interaction? For example, is there a gene-diet interaction in the Indian population?
- What is the role of macro- and micronutrients in Indian Diet?
- Do rural-urban migrants have increased diabetes and obesity?
- Does the pattern of migration influence risk?
- What are the potential interventions to reduce the risk?
- Finally, is there an evolutionary basis for the increased risk of diabetes^30^.

## How to answer these questions: The need for research and capacity development

The answer to these questions lies in trans-disciplinary research involving basic science, epidemiology, health systems, health economics and health policy disciplines. More importantly, operational research to implement the evidence based strategies generated from INTERHEART is critical in providing access to rural communities with poor referral linkages. Emphasis on upstream interventions such as policy level changes and research to identify barriers in prevention are other research priorities.

Considering higher levels of risk factors in Indians even among younger ages, developing innovative strategies incorporating both high-risk and population-wide strategies are crucial to prevention. Therefore, trials investigating the effects of a fixed-dose, combination therapy (Polypill) in various population groups in India and estimating its cost-effectiveness is highly justified.

## Conclusion

INTERHEART study results highlight the role of potentially modifiable risk factors in causation of acute myocardial infarction. These results suggest the feasibility of low cost prevention strategies to address the growing threat of chronic diseases in India.
